# A Self-Powered Nanogenerator for the Electrical Protection of Integrated Circuits from Trace Amounts of Liquid

**DOI:** 10.1007/s40820-019-0338-1

**Published:** 2019-12-14

**Authors:** Zhuang Hui, Ming Xiao, Daozhi Shen, Jiayun Feng, Peng Peng, Yangai Liu, Walter W. Duley, Y. Norman Zhou

**Affiliations:** 1 Beijing Key Laboratory of Materials Utilization of Nonmetallic Minerals and Solid Wastes, National Laboratory of Mineral Materials, School of Materials Science and Technology, China University of Geosciences, Beijing, 100083 People’s Republic of China; 2 Department of Mechanics and Mechatronics Engineering, Centre for Advanced Materials Joining, University of Waterloo, Waterloo, ON N2L 3G1 Canada; 3 School of Mechanical Engineering and Automation, Beihang University, Beijing, 100191 People’s Republic of China; 4 Waterloo Institute of Nanotechnology, University of Waterloo, Waterloo, ON N2L 3G1 Canada; 5 Institute for Quantum Computing, University of Waterloo, Waterloo, ON N2L 3G1 Canada; 6 State Key Laboratory of Advanced Welding and Joining, Harbin Institute of Technology, Harbin, 150001 People’s Republic of China; 7 Department of Physics and Astronomy, University of Waterloo, Waterloo, ON N2L 3G1 Canada

**Keywords:** Self-powered generator, Sub-millisecond response, Liquid protection

## Abstract

With the increase in the use of electronic devices in many different environments, a need has arisen for an easily implemented method for the rapid, sensitive detection of liquids in the vicinity of electronic components. In this work, a high-performance power generator that combines carbon nanoparticles and TiO_2_ nanowires has been fabricated by sequential electrophoretic deposition (EPD). The open-circuit voltage and short-circuit current of a single generator are found to exceed 0.7 V and 100 μA when 6 μL of water was applied. The generator is also found to have a stable and reproducible response to other liquids. An output voltage of 0.3 V was obtained after 244, 876, 931, and 184 μs, on exposure of the generator to 6 μL of water, ethanol, acetone, and methanol, respectively. The fast response time and high sensitivity to liquids show that the device has great potential for the detection of small quantities of liquid. In addition, the simple easily implemented sequential EPD method ensures the high mechanical strength of the device. This compact, reliable device provides a new method for the sensitive, rapid detection of extraneous liquids before they can impact the performance of electronic circuits, particularly those on printed circuit board.

**Electronic supplementary material:**

The online version of this article (10.1007/s40820-019-0338-1) contains supplementary material, which is available to authorized users.

## Highlights

- A power generator based on carbon nanoparticles and TiO_2_ nanowires was fabricated by sequential electrophoretic deposition.
- Benefit from the special structure of the carbon nanoparticle films, the generator exhibited an fast and reliable response to liquids.
- A possible system for printed circuit board protection using an array of power generators was proposed.

**Electronic supplementary material:**

The online version of this article (10.1007/s40820-019-0338-1) contains supplementary material, which is available to authorized users.

## Graphical abstract

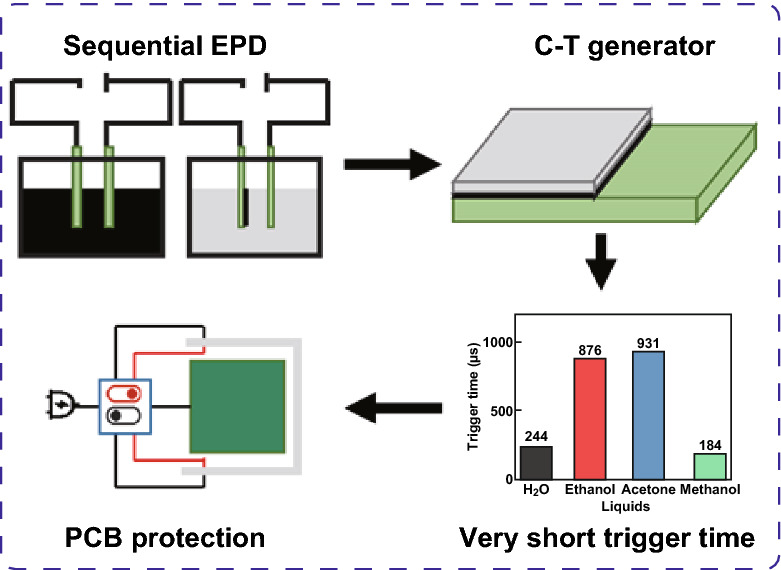

## Introduction

The laptop computer and other portable electronic devices have become indispensable tools in modern society, but all of these devices are susceptible to water damage. The most common source of water damage occurs due to seepage followed by the infiltration of liquid into printed circuit boards (PCBs). The failure mode involves the creation of a short circuit which compromises the functionality of individual components, producing large-scale damage to the overall device. Waterproofing technologies such as specialized packaging and the typical application of glue/rubber sealants have been utilized to protect laptops and other electronic devices from water and other liquids, but the packaging and sealing of PCBs are expensive and can decrease the dissipation of excess heat [1]. Recent research on water-induced power generators shows that the electrical output of these units can be sufficient to power external circuits [2–11], suggesting an alternative to solve this problem. This new methodology would involve using the electrical output of an onboard water-induced power generator to trigger the cutoff of power to the PCB prior to the formation of a short circuit induced by the ingress of liquid.

Various forms of carbon materials are commonly used in water-induced power generation devices, including carbon black [4, 12], porous carbon [13], carbon nanotubes [8], graphene [7], and graphene oxide [3, 5, 11]. These materials, generally applied as a deposited film, bond well to substrates making them suitable for applications where water resistance is required. However, these carbon materials typically exhibit slow response times which exclude applications involving fast switching. In addition, prior to operation as a power generator, pre-treatment and/or post-treatment is often necessary [3–5, 8, 13, 14].

Other studies have shown that matrices of one-dimensional (1D) TiO_2_ nanowires (NWs) used in water-induced power generation devices have several advantages compared to carbon-based materials because of their unique optoelectronic and geometrical properties and significantly faster response time to the adsorption of water [6, 9, 10, 14, 15]. TiO_2_ is an N-type semiconductor with a wide bandgap: anatase 3.2 eV and rutile 3.0 eV [16–18]. Under normal conditions, most of the electrons are in the valence band, and the conductivity increases only when the electron traps are ionized. The high resistance of TiO_2_ NWs makes them ideal for applications in water-induced power generators as this minimizes the possibility of an internal short circuit [6, 9, 10]. In addition, the large specific surface area and the super-hydrophilic surface of TiO_2_ NWs can greatly increase their sensitivity to the adsorption of liquids, facilitating their use as a medium for water-induced power generation [6, 9, 10, 14, 15]. However, the electrical response of power generators fabricated with TiO_2_ NWs is still not fast enough to permit the prompt detection of liquids [6, 9, 10].

In this report, we show that the performance limitations can be mitigated when the power generator is fabricated via the sequential deposition of carbon nanoparticles (CNPs) and TiO_2_ NWs on a titanium substrate (C-T). The resulting C-T generator is characterized by high open-circuit voltage and short-circuit current as well as a high sensitivity to trace amounts of water. The sensitivity to small quantities of ethanol, methanol, and acetone was also studied. In all cases, the response time is less than 0.1 s, indicating that devices incorporating this new C-T composition will be useful for the real-time detection of liquid infiltration on PCBs and the dynamic protection of circuit components.

## Experimental Methods

### Synthesis of TiO_2_ NWs

TiO_2_ NWs were synthesized using a modified hydrothermal method. Two grams of P25 was dispersed in 60 mL of NaOH alkaline solution (10 M) in a 125-mL Teflon-lined stainless steel autoclave and kept in a furnace maintained at 250 °C for 24 h. After cooling to room temperature, the suspended nanowires were taken out and washed with deionized water until the washing solution was neutral. Subsequently, the nanowires were immersed in 100 mL of HCl solution (0.1 M) and kept for 12 h to permit ion exchange. The solution was then filtered and washed again with deionized water. The products were collected and dried in an oven at 80 °C for 12 h. The resulting dried powder was then annealed in a furnace at 700 °C for 2 h to produce TiO_2_ NWs.

### Sequential EPD of C-T Generator Material

Hundred milligrams of Mg(NO_3_)_2_ was first dissolved in 200 mL of anhydrous ethanol, and then, 100 mg of CNPs was added to the resulting solution. The mixed solutions were then strongly stirred to ensure that the solid was fully dispersed.

For the EPD of TiO_2_ NWs, 0.25 g of TiO_2_ NWs was dispersed in a solution consisting of 125 mL of anhydrous ethanol, 13 mg of iodine, 0.2 mL of acetylacetone, 2 mL of acetone, and 1 mL of water. Before deposition, the mixture was strongly stirred to ensure that the solid was dispersed uniformly.

**Fig. 1:**
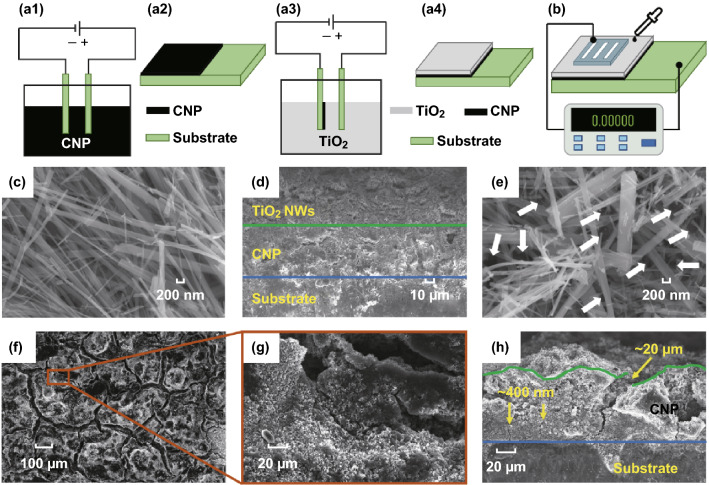
**a** Schematic showing the relevant steps in the sequential deposition of the CNP films and the TiO_2_ NWs onto a Ti electrode. **b** Measurement of open-circuit voltage and short-circuit current. **c** SEM image of as-synthesized TiO_2_ NWs. **d** A cross-sectional view of the films deposited on the Ti electrode. The Ti-CNP boundary is indicated with the blue line, while the CNP-TiO_2_ NWs boundary is shown as the green line. **e** Morphology of the top surface of the TiO_2_ NWs film in the C-T generator. **f** The top surface of a CNP film after a 4-min deposition. **g** Magnified image of the selected area in **f**. **h** A cross-sectional view of the CNP films. (Color figure online)

Figure 1a1–a4 shows a schematic representation of the fabrication of a C-T generator by sequential EPD. Two titanium sheets were ultrasonically cleaned in sequence in acetone, ethanol, and water before use. The clean titanium sheets were separated by 3 mm and oriented parallel to each other in the colloidal solution where they acted as electrodes. As shown in Fig. 1a1, a CNP film was first deposited on one titanium sheet using a direct current (DC) voltage of 30 V applied across the electrodes. The DC bias causes the charged CNPs to migrate toward the cathode, where they are deposited to form a uniform thin film (Fig. 1a2). The electrode with the adherent CNP film was then dried in an oven at 80 °C for 30 min to remove any residual ethanol prior to the deposition of TiO_2_ NWs on top of the CNP layer. As shown in Fig. 1a3, the C-T generator was fabricated by subsequent deposition of a TiO_2_ NWs film on the CNP film. The C-T generator was then placed in an oven at 100 °C for 12 h to remove ethanol. The deposition voltage used in TiO_2_ NWs film deposition was 30 V. Figure 1a4 shows a schematic diagram of the final configuration of the C-T power generator following sequential deposition.

### Material Characterization and Measurement of Power Generation

The morphology of the TiO_2_ NWs, CNP film, and the C-T generator were examined by scanning electron microscopy (SEM, Zeiss Ultra Plus. EHT: 10 kV, Signal: InLens). A zeta potential analyzer (Wallis) was used to measure the zeta potential of CNPs and TiO_2_ NWs. The open-circuit voltage (*U*_OC_) and short-circuit current (*I*_SC_) were recorded with a digital multimeter (Agilent 34401A). An oscilloscope (Keysight DSOX2012A) was used to record the fast output response (*U*_OC_) of the C-T device on exposure to a liquid. Figure 1b shows the setup of the electrical performance test with a 10 × 10 mm^2^ aluminum mask used as a top electrode on the C-T generator.

## Results and Discussion

An SEM image of as-synthesized TiO_2_ NWs is shown in Fig. 1c. The TiO_2_ NWs have a smooth surface and a diameter of ~ 100 nm. Figure 1d shows a cross-sectional SEM image of the sandwich structure of the C-T generator as fabricated by sequential EPD. The green line in the SEM image of the C-T generator indicates the boundary between the TiO_2_ NWs and the CNP films. The boundary between the CNP film and the titanium substrate is indicated by the blue line. A magnified image of the top-TiO_2_ NWs film on the C-T generator is shown in Fig. 1e. A comparison with the structure of an as-synthesized TiO_2_ NWs film as shown in Fig. 1c indicates that the morphology of the TiO_2_ NWs network deposited onto the CNP film is the same as that of the native TiO_2_ NWs material. The TiO_2_ NWs network deposited on the CNP film contains an abundance of interstitial nanochannels (marked by white arrows). These nanochannels provide conduits for liquids flow into the device and enable the mechanism that enhances the output voltage of the C-T generator [9, 14].

SEM images of the CNP films after EPD show that the surface is broken into a series of small structures separated by microscale cracks with width about 20 μm (Fig. 1f). Higher-resolution image reveals that sub-micron cracks with width about 400 nm are also present (Fig. 1g, h). The microscale cracks are likely formed during the drying process and propagate well inside the CNP film (Fig. 1h). The cracks arise because the normal stress imposed by the solvent through interfacial tension induces transverse tensile stress in the plane of the film that exceeds the strength of the close-packed network of particles bonded to the substrate during formation of the film [19]. The small size of the CNPs, compared to that of the TiO_2_ NWs, results in a denser CNP film on the substrate. It is apparent that the formation of cracks during the drying process creates abundant micro-/nanochannels that facilitate liquid diffusion. During EPD of TiO_2_ NWs, the NWs preferentially fill the cracks in the surface of the CNP film. This enhances bonding between the TiO_2_ NWs and CNP films. These properties are responsible for the fast adsorption rate for liquids and the rapid electrical response of the C-T generator.

**Fig. 2:**
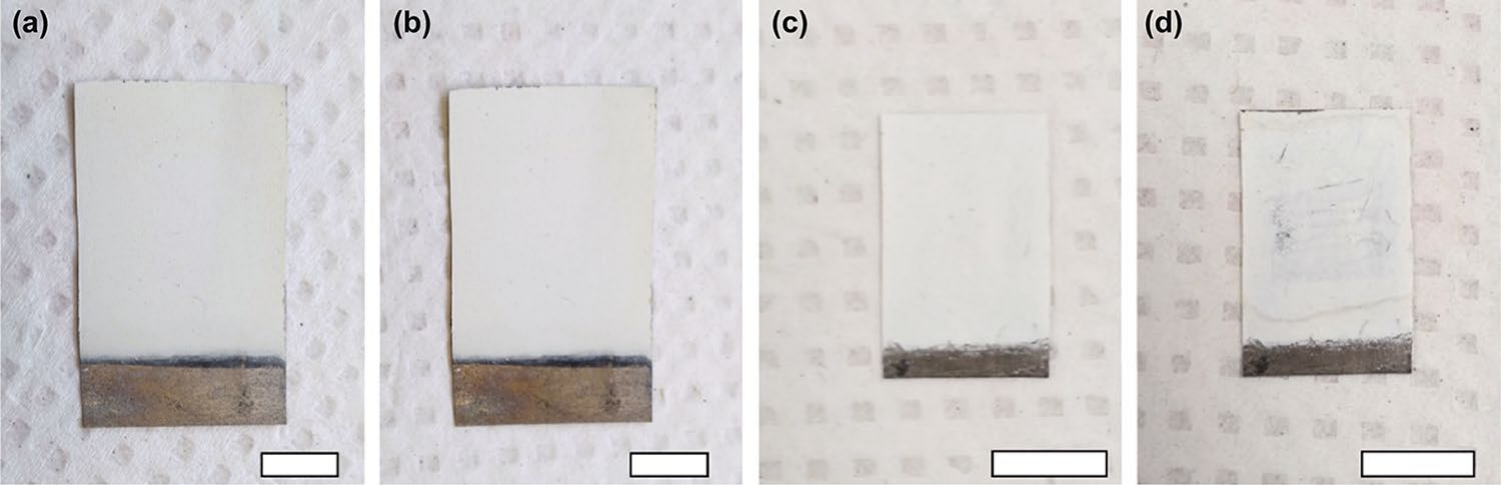
Digital images of the C-T generator: **a** before test, **b** after test. Similar images for the TiO_2_ NWs generator: **c** before test, **d** after test. The scale bar is 1 cm

Digital images of the upper surface of the C-T and the TiO_2_ NWs generators before and after the water drop test are shown in Fig. 2. The fabrication method used for the TiO_2_ NWs generator was basically same as that for the C-T generator, except that no EPD formation of CNP film. Before the test, the top surface of each generator is intact and clean (Fig. 2a, c). After dropping water on the top surface, it is apparent that the structure of the C-T generator remains unchangeable, indicating that the device is resistant to water damage. However, some loss of integrity is observed on the upper surface of the TiO_2_ NWs generator after the test (Fig. 2d). These results indicate that the bond between the CNP and the TiO_2_ NWs films enhances water resistance, which is essential application in the protection of PCBs from water infiltration.

**Fig. 3:**
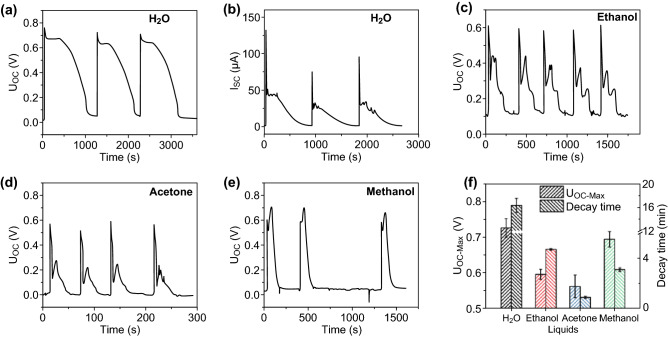
**a** *U*_OC_, **b** *I*_SC_ response of the C-T generator when 6 µL of liquid water is dropped on the device. After *U*_OC_ measurement, the C-T generator was left in the ambience to dry, followed by the *I*_SC_ test. The voltage response of the C-T generator to similar quantities of liquid ethanol, acetone, and methanol is shown in **c**–**e**, respectively. **f** Maximum *U*_OC_ and decay time for the C-T generator on exposure to different liquids

The time dependence of the voltage and current generated by the C-T generator when it contacts with a droplet of liquid water is shown in Fig. 3a, b. The response on exposure to a 6 μL water droplet is characterized by an intense sharp initial peak in both *U*_OC_ and *I*_SC_ indicating that the C-T generator is highly sensitive to liquid water. The initial sharp peak is followed by a lower amplitude output that remains constant over ~ 5 min. The combined output of the initial and secondary pulses typically lasts for ~ 16 min (Fig. 3a). Eventually, the output of the C-T generator is reduced to zero when water is evaporated completely from the device. Figure 3b shows that *I*_SC_ follows the same time-dependent response as *U*_OC_, but the peaks are much more pronounced. Repeating these measurements with other liquids yields a similar, but not identical, response. For example, when a 6 μL of ethanol was dropped on the C-T generator, *U*_OC_ initially increases to 0.60 (± 0.014) V and falls to ~ 0.31 V after about 10 s and then recovers to ~ 0.43 V which is maintained for about 5 min (Fig. 3c). The same volume of acetone introduced into the C-T generator initially gives *U*_OC_ of 0.56 (± 0.032) V falling to 0.14 V before recovering to ~ 0.28 V. The output voltage then gradually decreases to zero over ~ 30 s. With 6 µL of methanol, *U*_OC_ initially rises to 0.60 V and then follows by a higher amplitude peak with *U*_OC_ of 0.69 (± 0.022) V. After peaking at this voltage, *U*_OC_ decays back to zero (Fig. 3e). A summary of data for the different liquids is given in Fig. 3f. The different long-term decay times can be associated with the difference in evaporation rates of the liquids from the TiO_2_ NWs-CNP layers in the C-T generator [14] as the relative evaporation rate from the surface of the C-T detector depends on the adsorption energy and will be generally different from that of the pure liquid. Variations in the overall C-T response for different compositions suggest that this device will be useful for the detection of liquids in a variety of chemical environments.

**Fig. 4:**
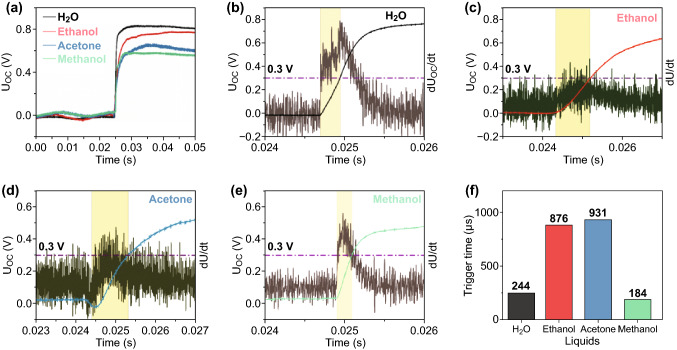
**a** Time dependence of the voltage response of the C-T generator for several liquids recorded with a fast oscilloscope (data acquisition frequency 1 MHz). Black, red, blue, and green curves response to H_2_O, ethanol, acetone, and methanol. Expanded *U*_OC_(*t*) plot of the initial response to droplets of H_2_O, ethanol, acetone, and methanol is shown in **b**–**e**. The purple curve in each figure plots d*U*_OC_/d*t*, while the trigger voltage was set to 0.3 V. **f** Effective trigger time for each liquid, defined as the time that the open-circuit voltage reaches 0.3 V. (Color figure online)

Expanding the time scale on the leading edge of *U*_OC_ shown in Fig. 3a indicates that the measured response time, defined as the time required to reach maximum *U*_OC_ = 0.73 (± 0.026) V, is 44.9 ms (Fig. S1). This response time is limited by the data acquisition rate of the multimeter. In order to evaluate the true response time of the C-T generator exposed to different liquids, a 6 µL droplet of liquid was placed on the detector and *U*_OC_ was monitored with an oscilloscope having a 1 MHz data acquisition rate with the trigger voltage set at *U*_OC_ = 0.3 V. Figure 4 shows the time-dependent response of the C-T generator as recorded at a 1 MHz data acquisition rate. The maximum value of *U*_OC_ in these traces is basically the same as that obtained using the digital multimeter. The response time of the C-T generator to water, ethanol, acetone, and methanol derived from *U*_OC_(*t*) is 244, 876, 931, and 184 μs, respectively (Fig. 4b–e). The slope of the voltage curve, d*U*_OC_/d*t*, also peaks within 1 ms and may provide an alternative signal for initiation of a protective response.

**Fig. 5:**
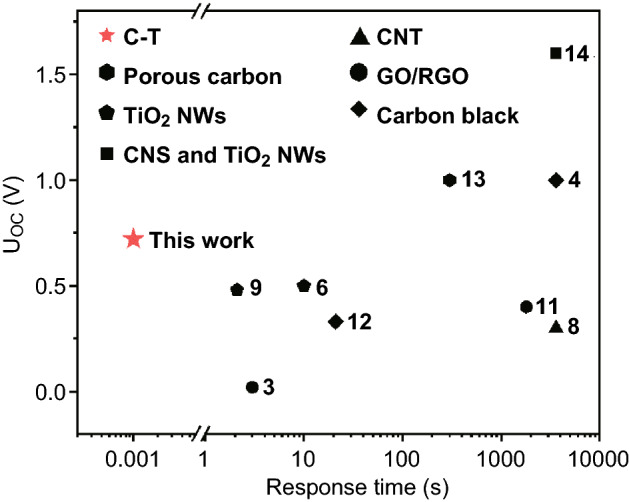
Comparison of the performance of the C-T power generator with that of other water-induced power generators

Figure 5 summarizes the power generation capability of the C-T generator in comparison with that of other water-induced power generators based on carbon black [4], GO/RGO [3, 11], CNT [8], porous carbon [13], TiO_2_ NWs [6, 9], and carbon nanospheres@TiO_2_ NWs [14] as reported elsewhere. It can be seen that only a few devices generate a *U*_OC_ more than 1.0 V. However, the response of these devices to water is quite slow and pre-/post-treatment such as exposure to a plasma [12], UV treatment [11], or high-temperature annealing (usually at about 375 °C for 150 min) [13, 14] are needed to functionalize these devices. The C-T generator in this work has been found to generate a *U*_OC_ more than 0.7 V without any pre-/post-treatment processing. In contrast to these other generators, the response of the present C-T device on exposure to liquids is typically less than 1 ms, which is three orders of magnitude faster than other devices. The detection limit for liquid water using the C-T generator was determined by dropping trace amounts of water on the generator. The *U*_OC_ response of the device to 1, 2, and 4 μL of water is shown in Fig. S2. The shape of the *U*(*t*)_OC_ curve generated by exposure to 1, 2, and 4 μL of water is similar to that obtained with 6 μL of water (Fig. 3a), indicating that the device is very sensitive to trace amounts of water.

We suggest that the appearance of a liquid induced voltage/current in the C-T device is due to the streaming potential, which arises when fluids subject to a pressure gradient flow through a narrow channel whose walls contain a surface charge [12, 20]. In this study, CNPs and as-synthesized TiO_2_ NWs are both negative charged [21, 22]. Their zeta potentials in neutral water are ≈ − 15 and ≈ − 23 mV, respectively (Fig. S3). A voltage arising from the streaming potential can also be generated in other liquids which are polarity protic solvents [14]. It has been determined that the flow of a polarity protic liquid in the micro-/nanochannels of a porous media causes flow currents due to the presence of an electric double layer at the interface between the polarity protic liquid and the channel walls [14]. During the diffusion of polarity protic liquids down a nanochannel, an electrical double layer is formed extending several nanometers out from the surface of the CNP and TiO_2_ NWs [6]. As individual molecules in the polarity protic liquid diffuse into channels having widths that are comparable to the width of the double layer, the flow of negative ions is impeded and only small positive ions (e.g., protons) can pass deeper into the channel. A separation of negative and positive ions is then produced by the capillary flow resulting in a charge imbalance that establishes an electric field along the channel from the bottom to the top electrode [12, 13]. It is evident that the highest values of *U*_OC_ in the C-T generator will be produced by high-polarity protic liquids such as water and methanol. Low-polarity protic liquids such as acetone and ethanol do not form a strong electric double layer and result in lower *U*_OC_ [12, 13].

**Fig. 6:**
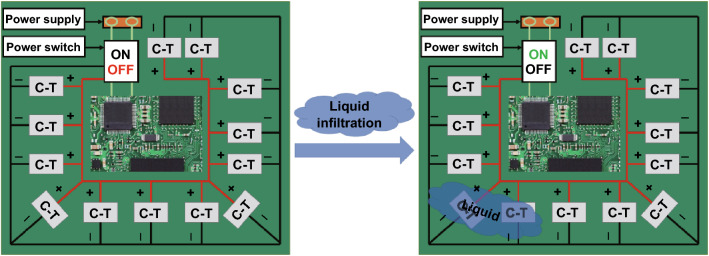
Schematic of a possible system for PCB protection using an array of C-T generators

Practical realization of PCB protection from liquids using as-prepared C-T generators could involve a configuration as shown in Fig. 6. The display, game, and other functions can only be realized when the work units are powered and work normally. In this protective configuration, an array of C-T generators would be mounted at locations on the PCB where liquid infiltration is anticipated to occur. These are connected as shown to a power switch. Under normal operating conditions without liquid infiltration, C-T generators are not triggered, the power switch is in the OFF state and the PCB is power supply providing the signals required for the operation of the protected electronics. When one or more C-T generators are triggered by the presence of a liquid, a voltage signal is generated in less than 1 ms that acts to turn ON the power switch that cuts off power to the PCB. This operation protects the circuits from damage. The response of the C-T generator was tested again after 6 months to characterize long-term reliability (Fig. S4). The *U*_OC_ output produced when 6 μL of water was dropped on the generator still shows a stable/fast response, indicating that the generator is reliable and that an extended shelf life is possible for these devices.

## Conclusions

A simple, easily implemented universal sequential EPD method was developed to fabricate a new type of composite CNP and TiO_2_ NWs power generator. The measured open-circuit voltage of this C-T device is 0.73 (± 0.026), 0.60 (± 0.014), 0.56 (± 0.032), and 0.69 (± 0.022) V on exposure to 6 µL of water, ethanol, acetone, and methanol, respectively. The presence of micro-/nanoscale-sized cracks on the CNP film ensures that the device has a high sensitivity to liquids and is not susceptible to water damage. The trigger time of the C-T generator is less than 1 ms and can be as small as 180 µs for certain liquids, which has never been achieved in water-induced power generation devices. Therefore, the as-prepared devices are promising candidates as sensors for a trace amount of liquids in applications that provide protection from short circuits in PCBs induced by the incursion of liquids. They are also suitable for applications involving power generation from ambient moisture.

## Electronic supplementary material

Below is the link to the electronic supplementary material.

Supplementary material 1 (PDF 385 kb)
