# Supplementary material for: A Self-Powered Nanogenerator for the Electrical Protection of Integrated Circuits from Trace Amounts of Liquid
Source: Nanomicro Lett. 2019 Dec 14;12:5. doi: 10.1007/s40820-019-0338-1 (PMC7770946; doi:10.1007/s40820-019-0338-1)
Supplement: Supplementary file 1 — Supplementary material 1 (PDF 385 kb) [file 40820_2019_338_MOESM1_ESM.pdf]

Supporting Information for

A Self-Powered Nanogenerator for the Electrical Protection of Integrated Circuits from Trace Amounts of Liquid

Zhuang Hui[1, 2, 3], Ming Xiao[2, 4], Daozhi Shen[5], Jiayun Feng[2, 6], Peng Peng[3], Yangai Liu[1, *], Walter W. Duley [2, 7], Y. Norman Zhou[2, 4]

[1]Beijing Key Laboratory of Materials Utilization of Nonmetallic Minerals and Solid Wastes, National Laboratory of Mineral Materials, School of Materials Science and Technology, China University of Geosciences, Beijing 100083, People's Republic of China

[2]Centre for Advanced Materials Joining, Department of Mechanics and Mechatronics Engineering, University of Waterloo, Waterloo, Ontario N2L 3G1, Canada

[3]School of Mechanical Engineering and Automation, Beihang University, Beijing 100191, People's Republic of China

[4]Waterloo Institute of Nanotechnology, University of Waterloo, Waterloo, Ontario N2L 3G1, Canada

[5]Institute for Quantum Computing, University of Waterloo, Waterloo, Ontario N2L 3G1, Canada

[6]State Key Laboratory of Advanced Welding and Joining, Harbin Institute of Technology, Harbin 150001, People's Republic of China

[7]Department of Physics and Astronomy, University of Waterloo, Waterloo, Ontario N2L 3G1, Canada

*Corresponding author. E-mail: liuyang@cugb.edu.cn (Yangai Liu)

Supplementary Figures

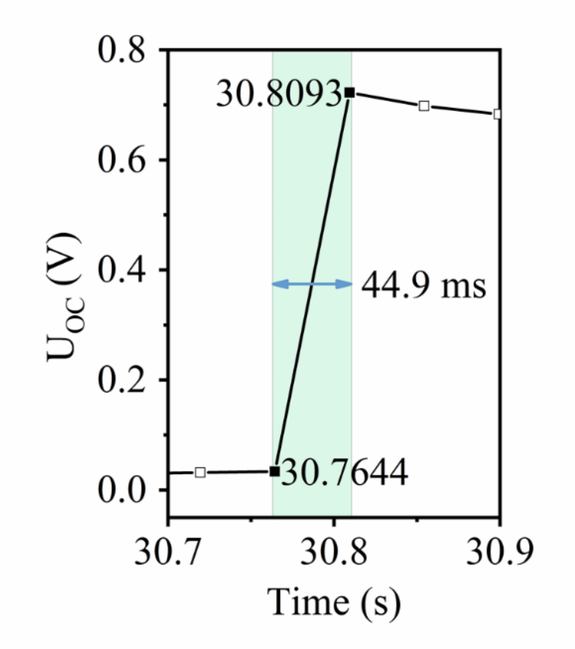

Fig. S1 The initial U_{OC} response in Fig. 3a at higher time resolution as measured with the multimeter. Note that the response time is limited by the intrinsic bandwidth of the multimeter

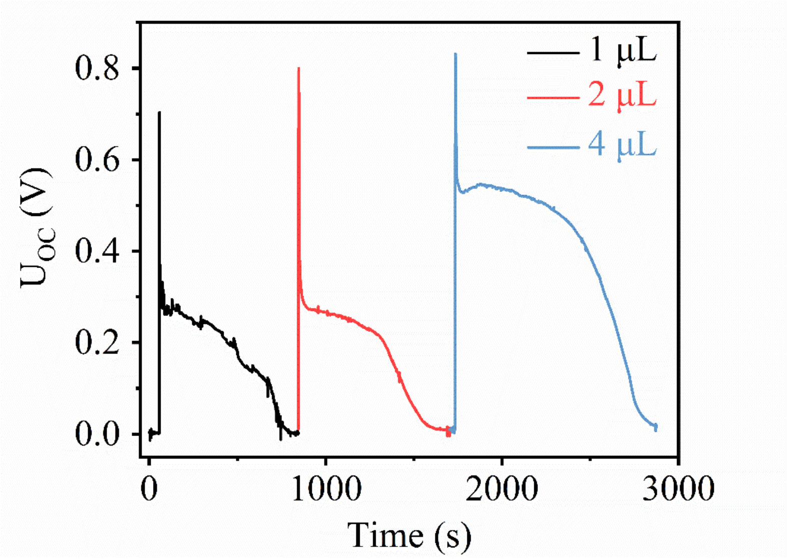

Fig. S2 The U_{OC} response of the C-T generator on dropping of 1, 2, and 4 μL of water

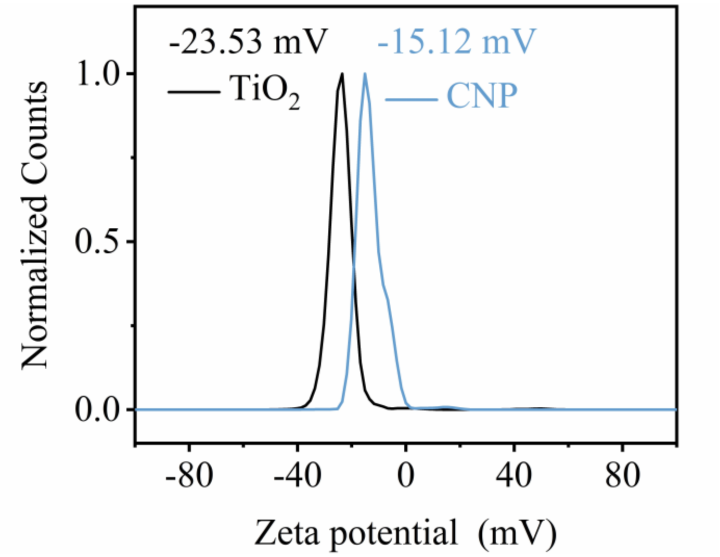

Fig. S3 Measured zeta potential for CNP and synthetic TiO_2 NWs dispersed in water with pH = 7

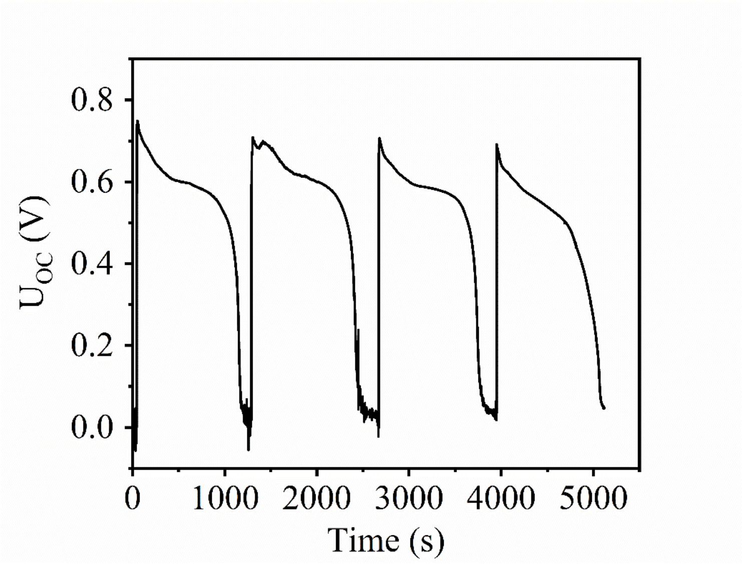

Fig. S4 U_{OC} response of the generator after a shelf life of six months
